# Shear strength characteristics of basalt fiber-reinforced loess

**DOI:** 10.1038/s41598-023-43238-z

**Published:** 2023-09-23

**Authors:** Chong-kun Chen, Gang Li, Jia Liu, Yu Xi, Jing-jing Nan

**Affiliations:** 1 Shaanxi Key Laboratory of Safety and Durability of Concrete Structures, Xijing University, Xi’an, 710123 Shaanxi China; 2 School of Geological Engineering and Geomatics, Chang’an University, Xi’an, 710054 Shaanxi China

**Keywords:** Solid Earth sciences, Natural hazards

## Abstract

Loess owns the characteristics of collapsibility, disintegration and solubility, which pose a challenge to engineering construction. To examine the shear strength of basalt fiber-reinforced (BFR) loess, consolidated undrained (CU) triaxial tests were conducted to explore the impacts of water content (*w*), fiber length (*FL*), fiber content (*FC*) and cell pressure (*σ*_3_) on the shear strength. According to the results, the shear strength model was established taken into account the impacts of *FL*, *FC*, and fiber diameter (*d*). The results showed that the peak strength of BFR soils enhanced as *FL*, *FC*, and *σ*_3_ increasing, whereas it decreased with increasing of *w*. Compared to unreinforced soil, the peak strength of BFR loess improved 64.60% when *FC* was 0.2% and *FL* was 16 mm. The optimum reinforcement condition for experimental loess was that of *FL* was 16 mm and *FC* was 0.8%. The reinforcing mechanism of fibers was divided into a single tensile effect and spatial mesh effect. The experimental and calculated results agreed well, which suggested the model is suitable for predicting the shear strength of BFR loess. The research results can offer a guideline for the application of BFR loess in the subgrade and slope engineering.

## Introduction

Loess is Quaternary sediment that is widespread in the northwest of China^1^. With the development of the "Belt and Road Initiative," modern transportation facilities represented by highways and high-speed railways have been built in large numbers^2–4^. However, the structural features of loess, such as porous, weakly cemented, and under compacted, lead to collapsibility, disintegration, and solubility, which pose a challenge to engineering construction^5^. Fiber reinforcement (FR) method provides an idea to solve engineering problems, and the fibers limit deformation of soil particles through the tensile force and frictional force, resulting in excellent mechanical properties of reinforced soil^6,7^. Ibraim et al.^8–10^ concluded that the compaction energy of loose fiber-reinforced sand is less than denser unreinforced sand when the peak strength keeps constant. The fiber-reinforced method can significantly reduced the liquefaction potential of sand in compression and extension loadings. A new sampling method for fiber-reinforced sand adopted vibration of moist sand/fiber mixtures was proposed and assessed. Reza Tabakouei et al.^11^ stated that the fiber type, fiber length, and specimen diameter determined the unconfined compression strength of fiber-reinforced sandy soil. Sharma and Kumar^12^ reported that the relative density remarkably affects the ultimate bearing capacity and settlement of fiber-reinforced sand, and the improvement effect reached maximum when relative density was 70%. Festugato et al.^13^ reported that inclusion of polypropylene fiber changed the dense sand stiffer than the unreinforced sand under cyclic loading. Choobbasti et al.^14^ concluded that polyvinyl alcohol fiber can improved the shear strength and axial strain at failure of Babolsar sand, while decreased the strength loess after peak strength. Soriano et al.^15^ discovered that the porosity of fiber-reinforced sand increased in the fiber vicinity, which validated the assumption of stolen void ratio. Mandolini et al.^16^ stated that the fiber strength governed by tensile strain domain and fiber orientation distribution.

For clayey soil, Abdi et al.^17^ concluded that polypropylene fiber can increased the compression, strength, and ductility of clay-lime composites. Hejazi et al.^18^ reported that the fiber content, fiber diameter, and fiber aspect ratio affected the shear strength of fiber-reinforced soil. Abbaspour et al.^19^ revealed that the waste tire textile fibers can improved the mechanical properties of expansive soil, and the swelling deformations was reduced by 44%. Consoli et al.^20,21^ reported that the ratio of porosity and cement played a critical role in evaluate the unconfined compression strength of fiber-reinforced soil–lime composites. Furthermore, the addition of fiberglass was ineffective to deduce the volumetric strain of fiber-reinforced sulfate-rich dispersive soil. Tamassoki et al.^22^ stated that 3% content of activated carbon and coir fiber can significantly improved the compressive strength, while 2% content can remarkably enhanced the shear strength of lateritic soil. Soleimani-Fard et al.^23^ revealed that discrete distributed fibers can significantly improved the shear strength, compressive, and hydraulic conductivity of fiber-reinforced fine-grained soil. Malekzadeh and Bilsel^24^ reported that the addition of polypropylene fiber can significantly decreased the swell-shrink of expansive soil, and shrinkage limit increased more than 50%. Phanikumar and Singla^25^ stated that the swell potential and swelling pressure of nylon fiber-reinforced expansive soil decreased with fiber length increased, and the secondary consolidation properties significantly enhanced for fiber-reinforced soil. Wang et al.^26^ concluded that the compressive and tensile strengths of collapsibility loess showed the trends of first increased then decreasing as increasing of glass fiber content (*FC*). Huang et al.^27^ found the FR can remarkably enhance the strength of remodeled loess. At the same time, the compressive modulus first increased then decreased with increasing *FC*, and the optimal *FC* was 0.6%. Xu et al.^28^ declared that the damage deviator stress of basalt FR (BFR) loess enhanced firstly and then reduced as *FC* increased, and the optimal *FC* was 0.6%. Zhu et al.^29^ found that the optimum condition for the unconfined compressive strength (UCS) of polypropylene FR loess with fiber length (*FL*) and *FC* was 12 mm and 0.5%, respectively. Meanwhile, the optimum condition for the deformation modulus was 12 mm *FL* and 0.3% *FC*. Zuo et al.^30^ adopted composite method to modify the soil, and they concluded that the compressive strength and flexibility of loess were effectively improved, and the optimal conditions were 1.5% of xanthan gum and 0.6% of basalt fiber. Lu et al.^31^ declared that the shear strength indices of polypropylene FR loess enlarged by 113.8% and 23.3%, respectively, whereas the disintegration rate decreased nearly 87.5%. An et al.^32^ observed the permeable ability of polypropylene FR soil increased significantly, and the protective effect of the loess slope was evident. Dong et al.^33^ found that the strength of lignin FR soil enhanced as cell pressure (*σ*_3_) increased, and the stress–strain curve transferred from hardening to softening with increasing *FC*. Chu et al.^34^ obtained that the strength of FR soil rose firstly, then reduced as increasing of *FC*, and the cohesion increased remarkably. Xiong et al.^35^ observed that the curves of BFR loess were converted from softening to hardening, and the shear strength indices were improved by 52.03% and 24.30%, respectively. Wang et al.^36^ concluded that basalt fibers can significantly improve the loess creep, and the creep deformation of BFR soils decreased with increasing *σ*_3_. Hu et al.^37^ noted the cohesion of FR loess enhanced firstly and subsequently reduced with increasing *FC*, and the optimal *FC* should be at least 0.2% in practical engineering. Gao et al.^38^ found that the UCS of samples prepared by the dilute mixing method was more suitable than that of the direct mixing method, and the effect of lignin *FC* on UCS was more obvious. Su and Lei^39^ pointed out that palm fiber can remarkably improve UCS of loess, and the influence of dry density on strength is significant, while the impact of *FL* is not significant. Chen et al.^40^ declared that the dynamic shear modulus of loess enlarged remarkably with raising fly ash content and cell pressure, whereas the damping ratio decreased as increasing fly ash content and *σ*_3_. Yang et al.^41^ found that the polypropylene fibers can change the cement-modified loess from brittle to plastic damage, and the fibers played a bridging role. The optimal reinforcement conditions were 0.30–0.45% of *FC* and 12 mm of *FL*.

Basalt fiber is a green inorganic composite material with high strength, temperature resistance, corrosion resistance, and no pollution, which has good application prospects in the fields of aerospace, manufacturing, and civil engineering. In order to validate the effective of basalt fiber-reinforced method, and establish a shear strength model of BFR loess. Based on consolidated undrained (CU) test, the effects of water content (*w*), *FL*, *FC*, and *σ*_3_ were analyzed, and the reinforced mechanism was revealed by loess microstructure used scanning electron microscopy (SEM). Furthermore, a shear strength model of BFR loess considering the effects of *FL*, *FC*, and fiber diameter (*d*) was established. The results can offer great guideline to applications of BFR soils.

## Materials and methods

### Experimental materials

The Loess was obtained from Yan'an (Shaanxi, China) in a construction site with a depth of 2.5 m, and Table 1 lists the basic parameters of loess. It can be found that experimental loess has the characteristics of low water content and large void ratio, which can be classified as silty clay. For engineering construction, loess foundation generally needs to solidify to improved the bearing capacity and reduce the settlement.

**Table 1:** Physical characteristics of loess.

Physical characteristics	Values
Specific gravity, *G*_s_	2.67
Dry density, *ρ*_d_ (g/cm^3^)	1.13
Water content, *w* (%)	9.00
Void ratio	0.83
Liquid limit, *w*_L_ (%)	25.00
Plastic limit, *w*_P_ (%)	12.00
Plasticity index, *I*_p_	13.00

Basalt fiber was bought from Shijiazhuang Zhuzhong Technology Co., Ltd (Hebei, China). The diameter and density of basalt fiber was 10 μm and 2.65 g/cm^3^, the tensile strength nearly 4000 MPa, and the elastic modulus reached 100 GPa.

### Sample preparation

During the sample preparation progress, the remolded loess was firstly smashed, and then sieved with a 2-mm griddle. The sample density was set as 1.45 g/cm^3^. The basalt fiber is disassembled into filiform, and the fixed length and content of fibers are evenly mixed with dry soil by electric blender to ensure the uniform distribution of fibers. Subsequently, a certain amount of water was adding to the blend fiber-reinforced soil and put in a glass container for 24 h. The sample size was 50 mm (diameter) × 100 mm (height), and it was produced by five levels.

### Experimental methodology

To investigate shear strength characteristics of BFR soil, the CU experiments were carried out by triaxial instrument. According to the standard for soil test method (GB/T 50,123–2019)^42^, tests were conducted at 0.5%/min strain rate, and were ceased at 20% axial strain. Table 2 lists the test program, 102 group experiments were carried out. The degree of saturation corresponded to 9% *w* and 13% *w* were 28.95% and 41.82%, respectively.

**Table 2:** Test program.

*σ*_3_ (kPa)	*w* (%)	*FL* (mm)	*FC* (%)
25	9, 13	0	0
25	9, 13	4	0.2, 0.4, 0.6, 0.8
25	9, 13	8	0.2, 0.4, 0.6, 0.8
25	9, 13	12	0.2, 0.4, 0.6, 0.8
25	9, 13	16	0.2, 0.4, 0.6, 0.8
50	9, 13	0	0
50	9, 13	4	0.2, 0.4, 0.6, 0.8
50	9, 13	8	0.2, 0.4, 0.6, 0.8
50	9, 13	12	0.2, 0.4, 0.6, 0.8
50	9, 13	16	0.2, 0.4, 0.6, 0.8
100	9, 13	0	0
100	9, 13	4	0.2, 0.4, 0.6, 0.8
100	9, 13	8	0.2, 0.4, 0.6, 0.8
100	9, 13	12	0.2, 0.4, 0.6, 0.8
100	9, 13	16	0.2, 0.4, 0.6, 0.8

## Results and discussion

### Stress–strain curves of BFR loess

#### Analysis of the Effect of *w*

**Figure 1:**
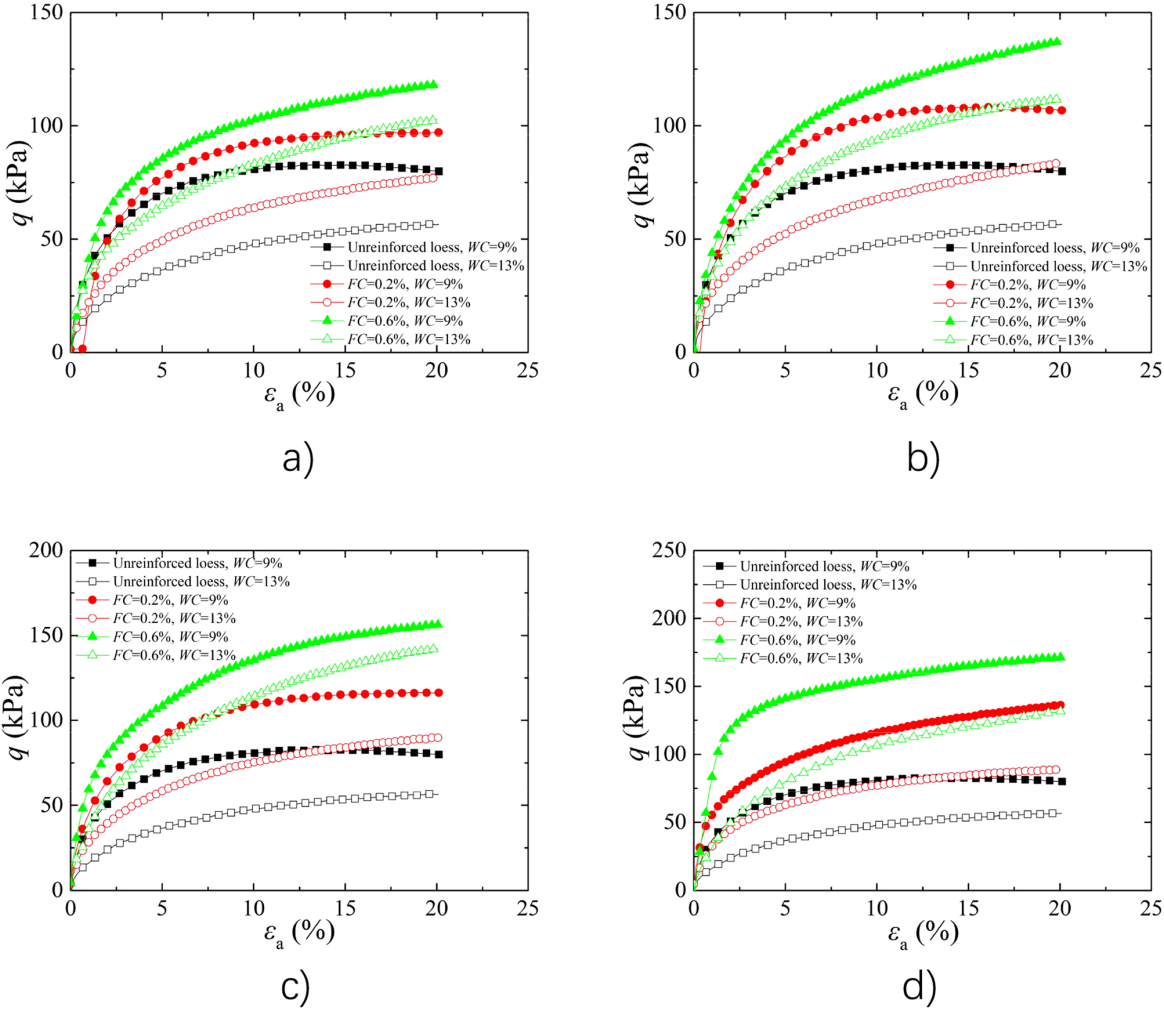
Effect of *w* on stress–strain curves (**a**) *FL* = 4 mm; (**b**) *FL* = 8 mm; (**c**) *FL* = 12 mm; (**d**) *FL* = 16 mm.

The water content had greater impact on mechanical characteristics of loess, while it impact on the FR soils deserves further study. Figure 1 shows the stress–strain curves of BFR soil with *FL* of 4 mm, 8 mm, 12 mm, and 16 mm under 25 kPa *σ*_3_. The peak deviator stress decreased with increasing *w*, and the peak strength of BFR loess is higher than that of loess, which consistent with reference^43^. For the unreinforced loess, the peak strength decreased by 31.65% at 13% *w* compared to 9% *w* under 4 mm *FL*. While for the BFR soils, the peak strength decreased by 20.38% and 12.93% at 13% *w* compared to 9% *w* when *FC* was 0.2% and 0.6%, respectively. The difference of deviatoric stress between 9 and 13% *w* at 0.2% *FC* was significantly larger than that of at 0.6% *FC*. The main reason is that fiber can improved the strength by limit the deformation of soil particles through the tensile force and frictional force, and the reinforcement effect at high *FC* was significantly larger than that of at low *FC*. Therefore, the difference of deviatoric stress between 9 and 13% *w* decreased with increasing *FC*.

#### Analysis of the effect of *FL*

**Figure 2:**
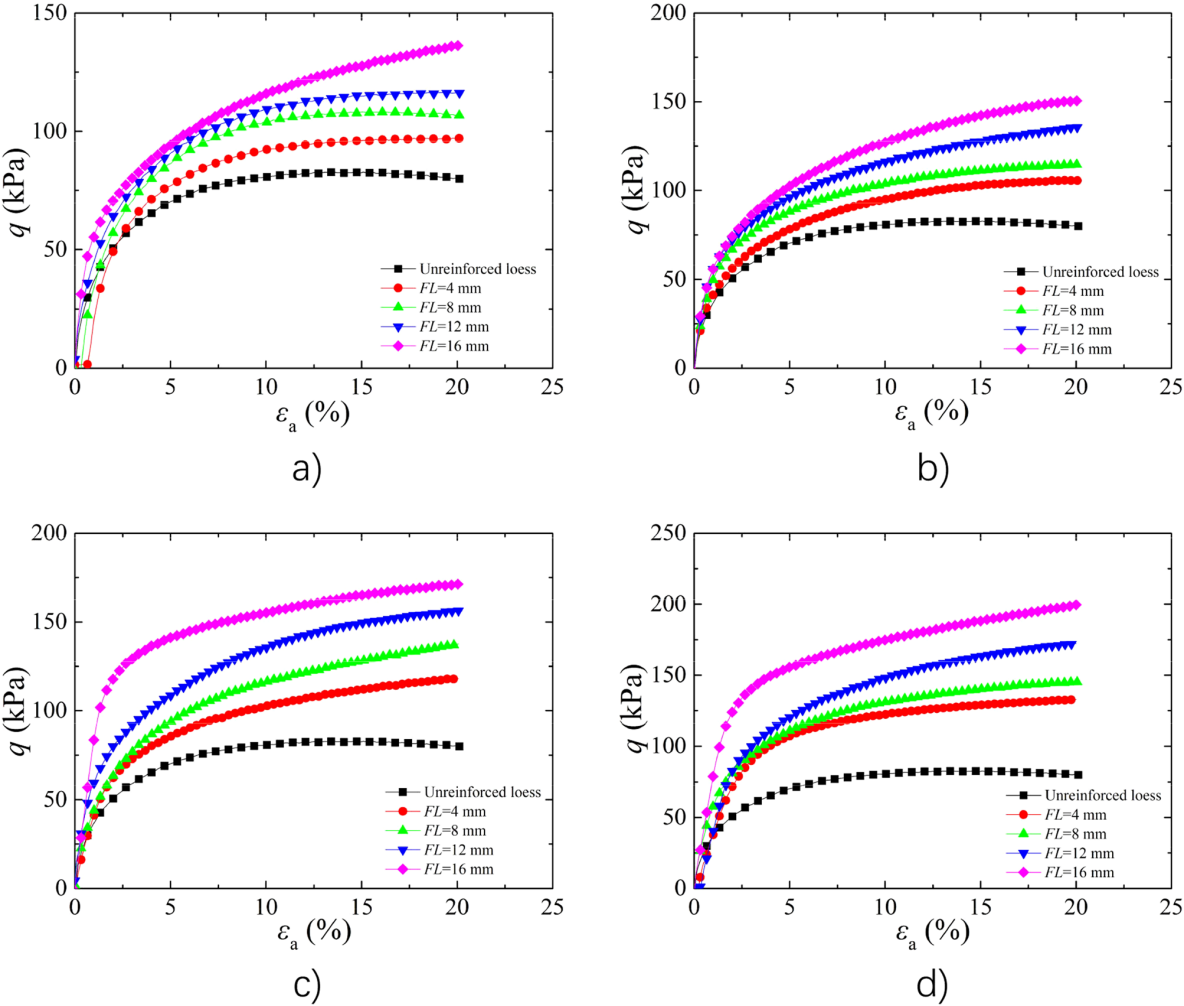
Effect of *FL* on stress–strain curves (**a**) *FC* = 0.2%; (**b**) *FC* = 0.4%; (**c**) *FC* = 0.6%; (**d**) *FC* = 0.8%.

The Fig. 2 shows the stress–strain curves of BFR soil with *w* of 9% and *FC* of 0.2%, 0.4%, 0.6%, and 0.8% under 25 kPa *σ*_3_. The curves of loess showed a strain-softening, whereas the BFR loess exhibited a strain-hardening^44^. The peak deviator stress of BFR soil increased as increasing of *FL*. Compared to unreinforced soil, the peak strength with *FC* of 0.2% and *FL* of 4 mm, 8 mm, 12 mm, and 16 mm increased by 17.21%, 28.97%, 40.45%, and 64.60%, respectively. The main reason is that fibers are randomly and uniformly distributed between the soil particles to form a composite that bears the loading together, resulting in significantly improved shear strength of BFR loess. As *FL* increased, the touch-points between fibers and soil particles increased results in the improvement of anchor effect. The restrain effect between the soil particles enhanced due to the stretching and flexible constraining by the fibers^45^.

#### Analysis of the Effect of* FC*

**Figure 3:**
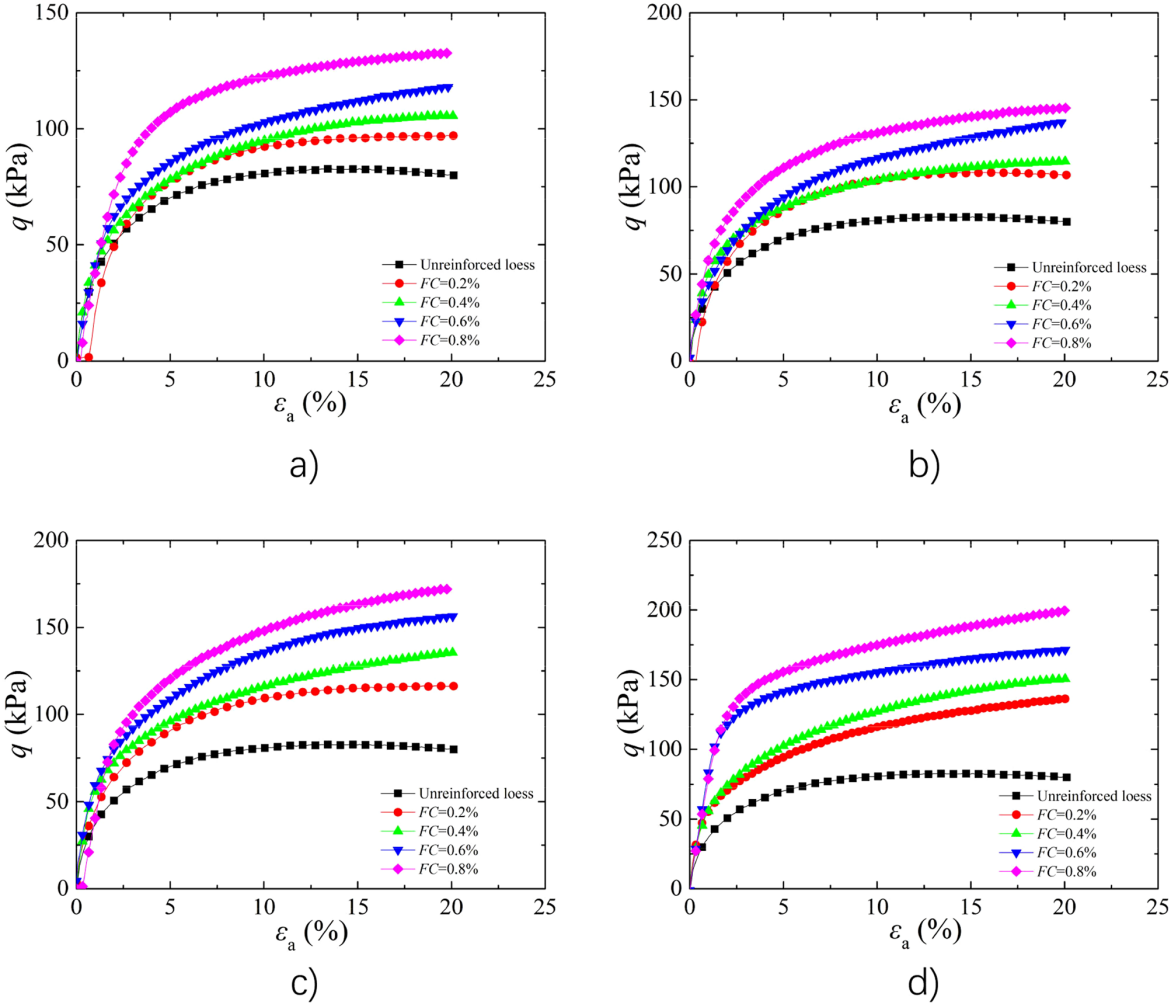
Effect of *FC* on the stress–strain curves (**a**) *FL* = 4 mm; (**b**) *FL* = 8 mm; (**c**) *FL* = 12 mm; (**d**) *FL* = 16 mm.

The Fig. 3 shows the stress–strain curves of BFR loess with *w* of 9% and *FL* of 4 mm, 8 mm, 12 mm, and 16 mm under 25 kPa *σ*_3_. The peak strength of BFR loess enhanced gradually by increasing *FC*, which is consistent with the results of references^46^. Compared to loess, the BFR soil with *FL* of 4 mm and *FC* of 0.2%, 0.4%, 0.6%, and 0.8%, the peak strength raised by 17.21%, 27.55%, 42.73%, and 60.66%, respectively. The main reason to explain the phenomenon is that with increasing of *FC*, the fiber number increased, which resulted in the more contact points between fiber and soil particles. Due to fibers can limit deformation of soil particles through anchoring effect, thereby result in improved the sample strength and prevent sample damage^22,23^.

#### Analysis of the Effect of ***σ***_3_

**Figure 4:**
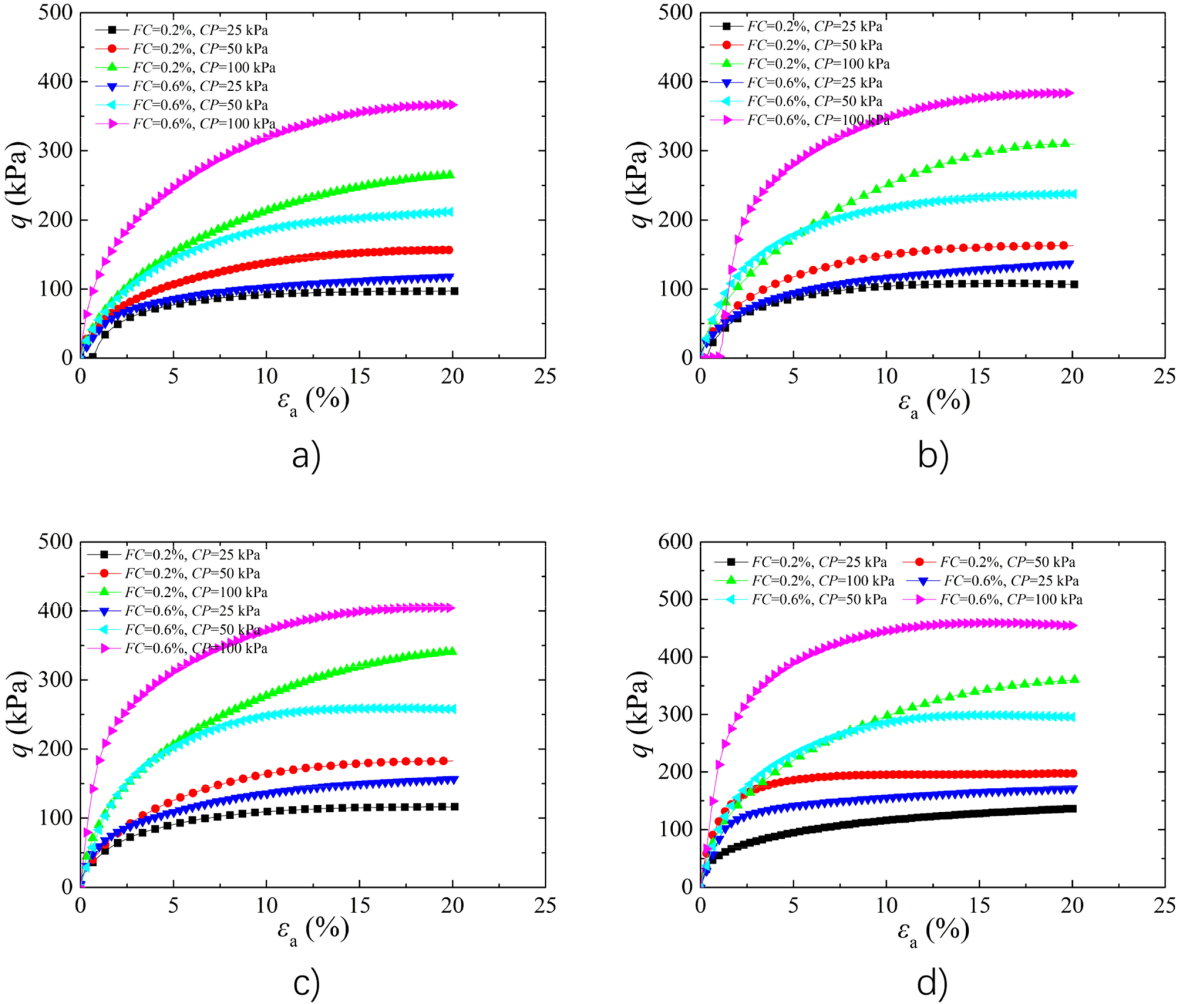
Effect of *σ*_3_ on the stress–strain curves (**a**) *FL* = 4 mm; (**b**) *FL* = 8 mm; (**c**) *FL* = 12 mm; (**d**) *FL* = 16 mm.

With embed depth increasing, the *σ*_3_ of the soil increased. Figure 4 shows the stress–strain curves of BFR loess with *w* of 9% and *FL* of 4 mm, 8 mm, 12 mm, and 16 mm. The peak strength increased with increasing *σ*_3_, indicating that the strength enhanced with embed depth, which is consistent with the results of the reference^47^. The BFR loess with *FL* and *FC* were 4 mm and 0.2%, the peak strength increased by 61.70% and 173.81% under 50 kPa and 100 kPa *σ*_3_, compared to that under 25 kPa *σ*_3_. Compared with the strength of BFR soil under 25 kPa *σ*_3_, the peak strength increased by 79.94% and 211.01% under 50 kPa and 100 kPa *σ*_3_ when the *FC* of 0.6%, respectively. The main reason is that as *σ*_3_ increased, the constrained of particles increased, and the anchoring effect of fibers by soil particles was enhanced, thereby the sample damage was prevented due to the high tensile strength of fibers, which resulted in the peak deviatoric stress increased and this is in agreement with the experimental results of cement-fiber treated sand^48^.

#### Microstructure characteristics of BFR loess

**Figure 5:**
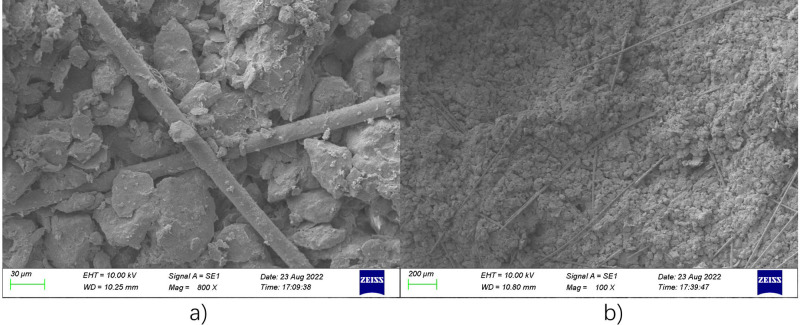
SEM pictures of BFR loess (**a**) Single tensile effect; (**b**) Spatial mesh structure.

The microstructure of BFR loess was measured by SEM as listed in Fig. 5. As shown in Fig. 5a, the fiber reinforcement is mainly based on the single tensile effect. The fiber was enveloped by a large number of soil particles. The mutual diastrophism was generated under shear loading result from high tensile strength. Furthermore, interfacial force was produced by the pullout of fibers, and mainly depends on the interfacial friction and adhesion. In the meantime, the restraining effect of fiber on soil particles was produced in the bending part when subject to pullout force, which limited soil deformation and improved shear strength. As shown in Fig. 5b, the fiber reinforcement is mainly based on the spatial mesh structure effect. Many fibers were randomly distributed and interwoven into a mesh structure. When one of the fibers is subject to a tension force, it pulls the other fibers to form a spatial force structure, resulting in the local loading transferred to a broader area, which further improves the tensile effect of the fibers^49^. In addition, Zhang et al.^50^ divided the reinforcement mechanism into bending mechanism and interweaving mechanism. The bending mechanism referred to fibers consisting numerous bends, almost no straight parts. When fiber bearing pullout loading, the friction were generated due to fiber bend. The interweaving mechanism referred to the interweaving points of fibers to form a spatial force structure to limit the displacement, and enhance the overall strength. Liu et al.^51^ concluded that the reinforcement mechanism resulted from interface strength, namely friction and cohesion. Furthermore, the interfacial friction primarily depends on the effect of particle shape, particle gradation, interfacial friction coefficient, and effective contact area. The interfacial cohesion mainly depends on the impact of clayey particles, the natural cement, and the interaction friction.

### Shear strength model of BFR loess

#### Model building

**Figure 6:**
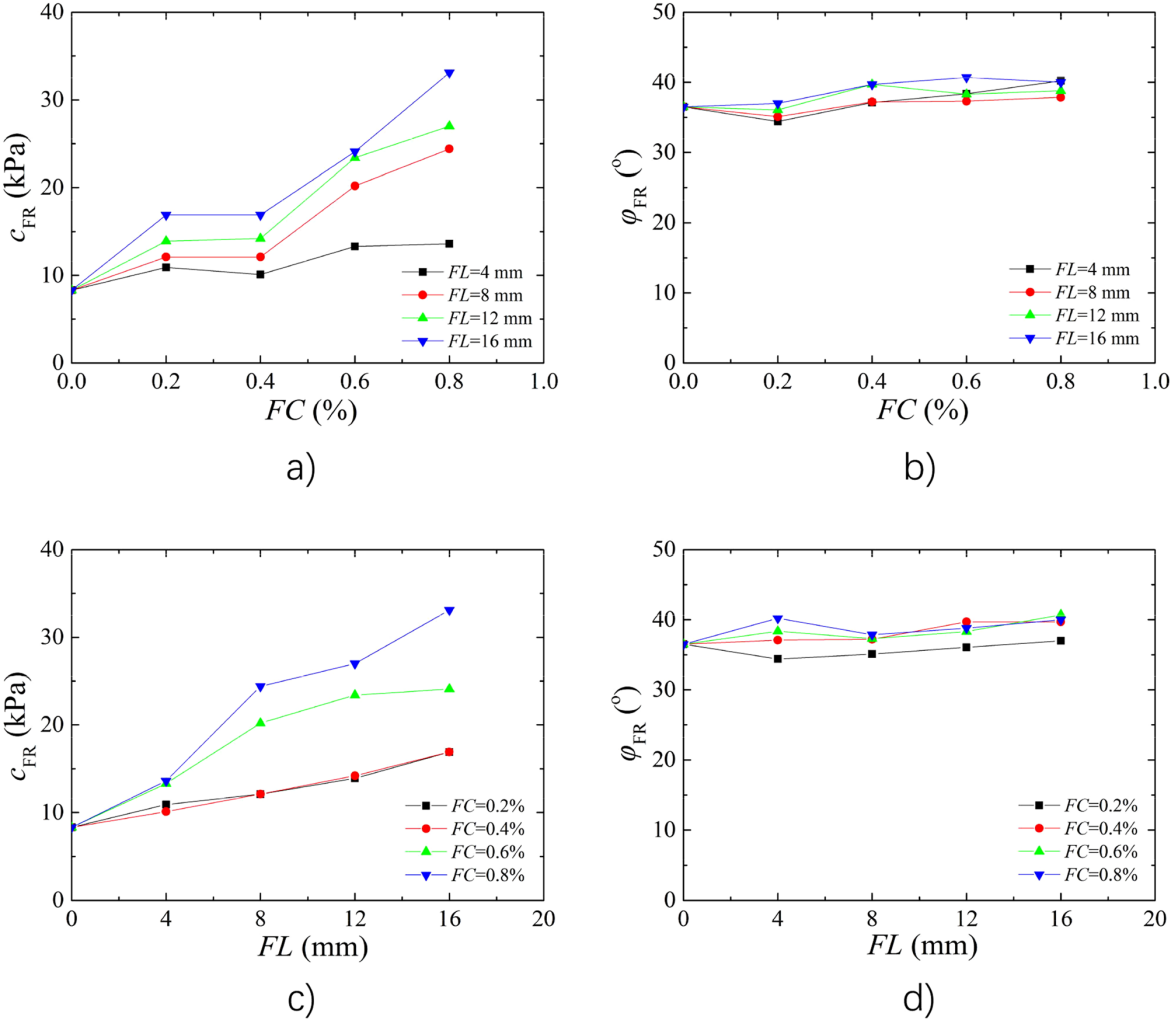
Effect of *FL* and *FC* on shear strength indices (**a**) Effect of *FL* on Cohesion; (**b**) Effect of *FL* on Internal friction angle; (**c**) Effect of *FC* on Cohesion; (**d**) Effect of *FC* on Internal friction angle.

The shear strength indices of unreinforced loess and BFR loess are summarized in Table 3. Figure 6a,b shows *FL* affect on the shear strength indices of BFR loess with *w* of 9%. The cohesion of reinforced soil increased as increasing of *FL*, while the internal friction angle changed insignificantly. When *FL* increased from 4 to 8 mm at 0.8% *FC*, the internal friction angle showed decreased trend. The main reason is that when the fiber bend distribution during the soil particles, the tensile strength and friction force cannot fully presented, thereby the internal friction angle may be decreased with increasing *FL*. When the *FC* of 0.8%, the cohesion of the BFR soil increased by 5.3 kPa, 16.1 kPa, 18.7 kPa, and 24.8 kPa with *FL* of 4 mm, 8 mm, 12 mm, and 16 mm, respectively. Figure 6c,d shows *FC* affect on the shear strength indices of BFR loess with *w* of 9%. On the whole, the cohesion of BFR soil increased with increasing *FC*, while internal friction angle varied little. The cohesion changed not significant between 0.2% and 0.4% *FC*, indicating that the reinforcement effect was not evident with low *FC*.

**Table 3:** Shear strength indices of BFR loess.

*w* (%)	*FL* (mm)	*FC* (%)	*C* (kPa)	*φ* (°)
9	0	0	8.3	36.5
9	4	0.2	10.9	34.4
9	4	0.4	10.1	37.1
9	4	0.6	13.3	38.4
9	4	0.8	13.6	40.2
9	8	0.2	12.1	35.1
9	8	0.4	12.1	37.2
9	8	0.6	20.2	37.3
9	8	0.8	24.4	37.8
9	12	0.2	13.9	36.1
9	12	0.4	14.2	39.7
9	12	0.6	23.4	38.3
9	12	0.8	27.0	38.8
9	16	0.2	16.9	37.0
9	16	0.4	16.9	39.7
9	16	0.6	24.1	40.7
9	16	0.8	33.1	40.0
13	0	0	6.2	31.8
13	4	0.2	6.7	31.5
13	4	0.4	8.9	31.2
13	4	0.6	10.0	33.4
13	4	0.8	12.6	33.5
13	8	0.2	7.1	31.8
13	8	0.4	10.1	32.6
13	8	0.6	12.6	34.1
13	8	0.8	16.5	33.4
13	12	0.2	8.4	32.0
13	12	0.4	15.2	28.8
13	12	0.6	17.4	33.1
13	12	0.8	19.4	34.2
13	16	0.2	9.3	32.1
13	16	0.4	18.0	30.9
13	16	0.6	18.4	33.8
13	16	0.8	20.8	35.4

The reinforcement mechanism of BFR loess is controlled by single tension effect and spatial structure, which formes a force transformation system to bear loading together. According to above research results and taken into account the dimensional effect, it is supposed that the cohesion of reinforced soil *c*_FR_ is a function of *FL*, and *FC* and *d*.

1 $$ c_{{{\text{FR}}}} = f\left( {\frac{FL \cdot FC}{d}} \right)c_{0} $$

where *c*_FR_ and* c*_0_ are cohesive of BFR loess and unreinforced loess, respectively, *FL* is the fiber length, *FC* is the fiber content, and *d* is the fiber diameter.

Equation (1) can be expressed as

2 $$ \frac{{c_{{{\text{FR}}}} }}{{c_{0} }} = f\left( {\frac{FL \cdot FC}{d}} \right) $$

By plotting the experimental results as curves of *c*_FR_/*c*_0_ versus FL*FC/*d*, it was found that *c*_FR_ /*c*_0_ tends to increase with increasing of FL*FC/*d*, and presented a nearly linear relationship, so it is assumed that

3 $$ c_{{{\text{FR}}}} = \left( {a + b \cdot \frac{FL \cdot FC}{d}} \right)c_{0} $$

where parameters *a* and *b* are the intercept and slope of the fitting curve.

Compared to unreinforced soil, the internal friction angle varied slightly with increasing *FL* and *FC*, so it is assumed that

4 $$ \tan \varphi_{{{\text{FR}}}} = \tan \varphi_{0} $$

where *φ*_FR_ and *φ*_0_ are the internal friction angle of BFR and unreinforced soils, respectively.

Based on Mohr–Coulomb theory, combined Eq. (1) with Eq. (4) gained

5 $$ \tau_{{{\text{FR}}}} = c_{{{\text{FR}}}} + \tan \left( {\varphi_{{{\text{FR}}}} } \right)\sigma $$

where *τ*_FR_ is the shear strength of BFR soil, and *σ* is the stress.

Taking Eq. (3) and Eq. (4) into Eq. (5), we obtained

6 $$ \tau_{{{\text{FR}}}} = \left( {a + b \cdot \frac{FL \cdot FC}{d}} \right)c_{0} + \tan \left( {\varphi_{{0}} } \right)\sigma $$

According to Eq. (6), when the parameters *c*_0_, *φ*_0_, *FL*, *FC*, and *d* are known, the unknown parameters (*a*, *b*) can be obtained using fitting method.

#### Fitting of model parameters

**Figure 7:**
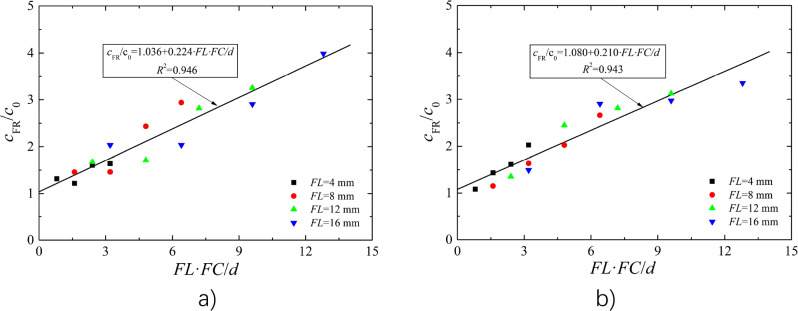
Fitting curves of cohesion (**a**) *w* = 9%; (**b**) *w* = 13%.

The Fig. 7 shows the fitting curves of cohesion of BFR loess with 9% and 13% *w*. The value of *c*_FR_/*c*_0_ linearly increased as the increasing of FL*FC/*d*, and the determined coefficient of the fitting curves reached 0.946 and 0.943 with 9%, 13% *w*, respectively, indicating that the correlation between the horizontal axis and vertical axis was good and can be expressed by a linear equation. Overall, considering of intercept *a* and slope *b* of the fitting curves with 9% and 13% *w*, the parameter values were determined as *a* = 1.0 and *b* = 0.2.

#### Model validation

**Figure 8:**
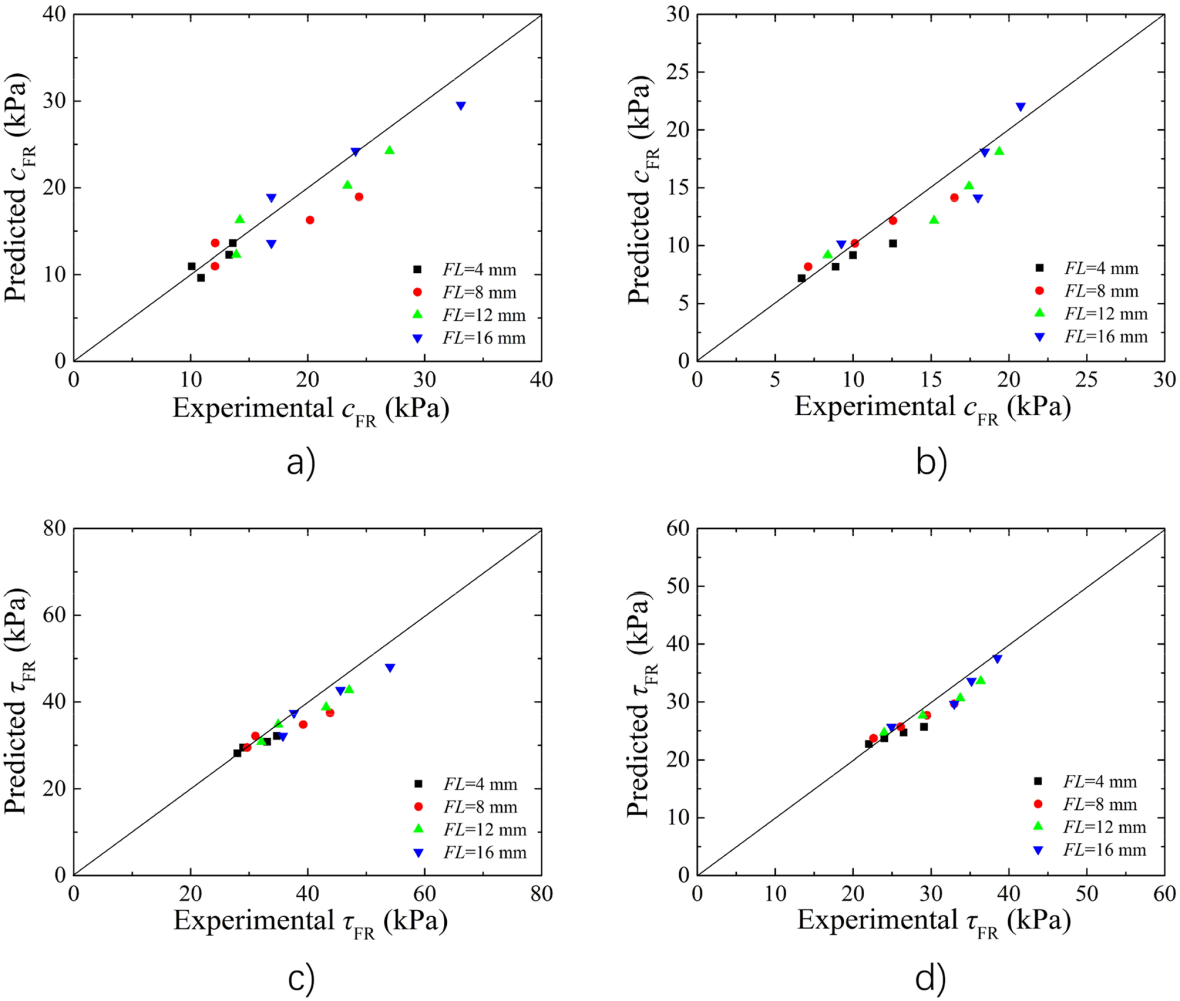
Comparison of the cohesion results and the shear strength results between measured and predicted (**a**) the cohesion results for *w* = 9%; (**b**) the cohesion results for *w* = 13%; (**c**) the shear strength results for *w* = 9%; (**d**) the shear strength results for *w* = 13%.

The parameter values of *c*_0_, *FL*, *FC*, and d were known, the cohesive of BFR loess *c*_FR_ can be obtained by substituting model parameters *a* and *b* into Eq. (3), and the *τ*_FR_ can be obtained by substituting parameters *a* and *b* into Eq. (6). Figure 8a,b shows the comparison of cohesion between experimental and predicted. It can be found that the cohesion data were relatively uniformly distributed on two sides of the parallels. Figure 8c,d shows the strength comparison of BFR soil between experimental and predicted. It can be found that the shear strength data were more concentrated and distributed on both sides of the parallels compared to the cohesive data, indicating that the shear strength predicted results agreed better with the experiment results. Based on the comparison of cohesion and shear strength, the predicted values and test values agreed well, which suggested that the model is suitable for predicting the cohesion and shear strength of BFR loess.

## Conclusions

According to consolidated undrained tests, the effects of water content (*w*), fiber length (*FL*), fiber content (*FC*), and cell pressure (*σ*_3_) on the shear strength of basalt fiber-reinforced (BFR) loess were investigated. The microstructure characteristics of BFR loess were constructed by SEM test to reveal the reinforcement mechanism of basalt fiber. Furthermore, a shear strength model considering fiber affects was established and verified. The main conclusions were drawn as bellow:

(1) The peak strength decreased with increasing *w*, and the BFR loess was remarkably modified compared to unreinforced loess. The peak strength decreased by 20.38% at 13% *w* compared to that at 9% *w* when *FC* was 0.2%.

(2) Loess showed strain-softening, whereas the BFR soils exhibited strain-hardening. With increasing *FL*, the peak strength of BFR soil increased. Compared to unreinforced soil, the peak strength of BFR loess increased by 17.21%, 28.97%, 40.45%, and 64.60% with *FC* was 0.2% and *FL* changed from 4 to 16 mm, respectively.

(3) With increasing *FC*, the peak strength of BFR soils gradually enhanced, and it increased with increasing *σ*_3_. When *FL* was 4 mm and *FC* varied from 0.2% to 0.8%, the peak strength raised by 17.21%, 27.55%, 42.73%, and 60.66%, respectively.

(4) The reinforcement mechanism was controlled by a single tension effect and spatial structure, which combined a force transformation system. When one fiber subject to a tension force, it pulls the other fibers to form a force transformation system, which further improves the overall tensile effect of the fibers.

(5) The optimum reinforcement condition for experimental loess was that of *FL* was 16 mm and *FC* was 0.8%. The predicted results and test results agreed well, which validated the dependability and indicated that the model is suitable to predict the shear strength of BFR loess.

## Data Availability

All data generated or analysed during this study are included in this published article.
